# Correction: Demographic and Clinico-Epidemiological Features of Dengue Fever in Faisalabad, Pakistan

**DOI:** 10.1371/journal.pone.0294300

**Published:** 2023-11-07

**Authors:** Eun Jeong Yu, Eun-A. Park, Seung-Ah Choe, Kyung-Ah Lee, You Shin Kim

The second author’s name is spelled incorrectly. The correct name is: Eun A. Park.

The following information is missing from the Funding statement: This study was supported by the National Research Foundation of Korea (NRF) grant funded by the Korean government (Ministry of Science and ICT, MSIT) [grant number 2021R1F1A1045993]
