# Supplementary material for: Freeze all-first versus biopsy-first: A retrospective analysis of frozen blastocyst transfer cycles with preimplantation genetic testing for aneuploidy
Source: PLoS One. 2022 Sep 26;17(9):e0267652. doi: 10.1371/journal.pone.0267652 (PMC9512210; doi:10.1371/journal.pone.0267652)
Supplement: Attachment — Submitted filename: response to reviewer.docx [file pone.0267652.s002.docx]

Reviewer #1: The authors of this manuscript have undertaken the task of analyzing retrospectively a considerable data set, in which two protocols in a single center dealing with the sequence of events in PGT-A were compared: (1) first freeze all blastocyst embryos, then thaw, then biopsy (n=211 couples, 232 blastocysts), or (2, the more common approach) first biopsy, then freeze all biopsied embryos (n=327 couples, 415 blastocysts). One or two embryos were transferred. The outcome of both protocols was analyzed up to ongoing pregnancy (12th week of gestation). Pregnancy outcome data were not provided. The authors conclude that the sequence of events, in which the embryos are first biopsied and then stored frozen is beneficial to the outcome of the treatment, as given by the ongoing pregnancy rate.

Having searched the literature, this is the first report dealing with this question. Given the clear-cut beneficial outcome of first biopsy the embryo before cryostorage, it is unlikely that a randomized control trial will ever be carried out (although suggested by the authors at the end of the discussion).

Both groups were not completely similar, as the basal FSH levels were higher in the first group, together with a somewhat higher mean BMI in the second group. It would certainly be appropriate to add some details on the decision processes in each of both study groups, perhaps integrated into Table 1 or in a separate Table.

The patients were treated in the time interval from Januar 2016 to Dezember 2019, but the follow-up included only ongoing pregnancies until week 12 of gestation. Most pregnancies must now have gone to delivery. Isn’t it possible to add all information about the outcome of the pregnancies, including the occurrence of complications during pregnancy and the status of the newborns?

-> We added your point to the table 1 and result section

The paper would benefit a lot from a thorough and careful edit by a native English speaker.

->We received English correction.

The authors might consider adding one reference (Cimadomo D, Rienzi L, Romanelli V, et al. Inconclusive chromosomal assessment after blastocyst biopsy: prevalence, causative factors and outcomes after re-biopsy and re-vitrification. A multicenter experience. Hum Reprod 2018;33:1839-1846), in which embryos with inconclusive results of genetic testing were subjected to thawing and re-biopsy. They provide some information about the feasibility of the (re)-biopsy of thawed embryos and their competence to become implanted after embryo transfer, as studied in this retrospective data set.

->Thanks for your suggestion. We added this article on result and reference section.(Line 266-268)

Figure 1: the authors should design the figure such, that the confidence intervals are visible.

->We added the confidence intervals on figure.

Table 1: Do the authors provide us with standard deviations, standard errors or 95% confidence intervals?

->We have corrected your point.

Line 74: replace the word “inevitable” by “irreparable”.

->We have corrected your point. (Line 76)

Line 97: all patients

->We have corrected your point.(Line 99)

Line 126: zona pellucida

->We have corrected your point.(Line 127)

Line 239: instead of (data not seen) please write (data not shown)

->We have corrected your point.(Line 270)

Discussion, lines 238 to 248: the authors might add some information about the influence of making a hole into the zona pellucida and the risk of monozygotic twinning.

-> we corrected what was pointed out

“Also, this change has been related with increased risk of monozygotic twin because of blstocyst herniation and embryo splitting through non-natural gap”(Line 279-281)

Reviewer #2: Freeze all-first versus biopsy-first: a retrospective analysis of frozen blastocyst transfer cycles with preimplantation genetic testing for aneuploidy

Manuscript by Jeong Yu et al., retrospectively compared the efficacy and safety of the timing on the cryopreservation and trophectoderm (TE) biopsy for PGT-A program. The main observation is increased clinical and ongoing pregnancy rates in biopsy-first approach than those in freeze all-first. Authors believe that performing TE biopsy of blastocyst for PGT-A before vitrification and warming can give better clinical outcome.

Major comments:

My major concern with this manuscript is lack of methodological details and importantly, rationale in comparing these two groups. There is no clarity in their objective. Even after reading the manuscript several times, I did not understand when embryo transfers were done in freeze- all first group, post warming and TE biopsy. Are the results available fast enough to perform ET in the same cycle, within a few hours? Or biopsied embryos were re-frozen? The methodology is not clear on this point. If re-freezing was done, then the survival rate and embryo quality of re-frozen/thawed embryos needs to be stated, and discussion on the safety of multiple freeze/thaw would be warranted. Also, if the embryos were re-frozen, the survival rate of ~98% in both groups is very interesting.

->Thanks for your comment. We tried to explain about this point on material and method part.

In the freeze-all first cycles, thawing blastocysts were prioritized based on the best quality before biopsy. Embryos were warmed in the early morning on the day before transfer, then cultured for 2-3h and assessed for TE biopsy. Biopsy results confirmed on the morning of scheduled transfer and transferred at least one euploid embryo. In biopsy-first cycles, once biopsy results confirmed at least one euploid embryo, patients were scheduled for a frozen ET cycle.(Line 169-174)

The author states, "We hypothesized that biopsy before cryopreservation may cause the embryo inevitable damage in the freezing process decreasing survival rates and implantation potential." Is it in comparison to biopsy after cryopreservation? The hypothesis has not been addressed in discussion, especially since the conclusion is contrary to the hypothesis.

->Thanks for your comment. We are sorry for confusion. We changed the paragraph from “biopsy before cryopreservation” to “biopsy after cryopreservation-thawing”(Line 75)

Was ICSI done for the freeze-all group too, when PGT was not planned at that point in time? If not, what measures were taken to avoid contamination during IVF to prevent false results in PGT.

->We used ICSI in all cases. ICSI is commonly used in pre-implantation genetic testing (PGT) cases to eliminate any risk of sperm DNA contamination.

Table 1: Mean age of patients is stated as 37.0±3.9 for freeze all first and 36.7±4.1 for biopsy first, but under indications for PGT, advanced age(>40yrs) shows 125(59.2%) and 222(67.8%), respectively. There appears to be some discrepancy in the data which needs to be addressed.

->Freeze all first group included more young patient with recurrent spontaneous abortion than biopsy first group.

Minor comments:

1. Line 67 - "there is no consensus regarding the timing of TE biopsy (before versus after cryopreservation)". Please cite reference.

-> We have corrected your point.(line 70)

Reference 8 : Aoyama N, Kato K. Trophectoderm biopsy for preimplantation genetic test and technical tips: A review. Reprod Med Biol. 2020;19(3):222-31.

2. Line 76 - does the "previous data" refer to the earlier reference? It is not clear.

->We changed the word from “data” to “study”. This refers to an article published prior to our study.(Line 77)

3. Ln 65-66 - reference given for this sentence has not been cited properly under references.

->We found new reference and change the reference(Line 66-67)

Greco E, Litwicka K, Minasi MG, Cursio E, Greco PF, Barillari P. Preimplantation Genetic Testing: Where We Are Today. Int J Mol Sci. 2020;21(12).

3. Ln 87 - " old age" can be rephrased as advanced maternal age and cut off can be mentioned. If any criteria were followed for the indications, please cite the references.

-> We have corrected your point and added the references. (line 89)

4. Line 127 - Blastocyst morphology ">3BC". The criteria used for grading can be stated; was 3BC the minimum grading to be eligible for biopsy? If so, it can be stated accordingly to avoid confusion.

-> We newly described about this point at the material and method part (line 128)

6. Line 131 - Details could be given regarding how the authors proceeded with the biopsy material, with a focus on duration of genetic analysis.

-> We newly described about this point at the material and method part (line 128-140)

7. Line 140 - Embryo transfer : How long after biopsy were the embryos transferred would require mentioning in the methodology section.

-> We newly described about this point at the material and method part (line 169-174)

8. Line 149/150 - "in biopsy-first cycles, where the blastocyst had not been subjected to assist hatching in the previous cycle, assisted hatching was performed" - are the authors referring to the freeze-all group? It's not clear.

-> This sentence corresponds to biopsy-first group.

9. Table 1 : Mean survival rate - It can be stated as mean survival rate of embryos

-> Thanks for your comment. We have corrected your point.

10. Line 238 - 242 not comprehensible in discussion, could be rephrased for better clarity.

-> Thanks for your comment. We have corrected your point.(Line 270-271)

“Hatching status could make significant different in clinical outcomes between biopsy-first and freeze all first groups.”

11. A further discussion on pregnancy outcomes & miscarriage rates between the two groups and also in comparing the findings with other studies would be warranted, since the study is looking into safety and efficacy of PGT timing.

->Thanks for your comment. We have corrected your point.(table and Line 205-208)

12. Spelling mistakes in a few places that need correction.

-> Thanks for your comment. We have corrected your point.
