# Supplementary material for: Freeze all-first versus biopsy-first: A retrospective analysis of frozen blastocyst transfer cycles with preimplantation genetic testing for aneuploidy
Source: PLoS One. 2022 Sep 26;17(9):e0267652. doi: 10.1371/journal.pone.0267652 (PMC9512210; doi:10.1371/journal.pone.0267652)
Supplement: Attachment — Submitted filename: response to reviewers_032322.docx [file pone.0267652.s003.docx]

Reviewer #1: The authors of this manuscript evaluate the effect of a freeze-all first strategy versus a biopsy-first strategy on the outcome of PGT-A in a cohort of more than 500 treatment cycles. The analysis was retrospective, but the strength of this study is that no other study has dealt with this topic so far. The paper has already undergone a major revision based up two extensive and high quality reviews. The quality of the manuscript has much improved, but a number of issues still remain.

As this is a retrospective analysis, it is important to explain why both strategies were used (freeze-all first versus biopsy-first). Between lines 87 and 93 some information is given, but not quantified. Especially the statement that poor ovarian reserve requiring repeated stimulations as a reason for freeze-all first is worrying, as this subpopulation is characterized by lower likelihood of pregnancy, thereby skewing the outcome of the study. This would also explain the slightly higher (but statistically significant) basal FSH-serum levels in the one study group. The authors should clearly state and quantify the reasons for selection into the one or into the other study group, either in the Material and Method section and/or in the Results section of their manuscript, as already requested by Reviewer 2.

- We added your point to the material and methods (line 89-94). As a result of reviewing the raw data again, there was no statistically significant difference between FSH and BMI.

One reviewer requested the authors to add live birth numbers to their results section. This was indeed partially done, but the authors should also implement Table 1 with these additional data. They should add the live birth numbers to the clinical pregnancy rate per ongoing ET, to ongoing pregnancy rate per ET and to miscarriage. The first reviewer also asked for data on incident complications during pregnancy and data on the newborn babies, but these data were not given (although stated to be given). Please, amend this valuable information, that will greatly benefit the quality of this study.

- We added your point to the table 1 and result section

The authors should also clarify in Table 1, whether the mean values are accompanied by standard error of the mean or by standard deviations.

- We added your point to the table 1 and result section

The authors have transferred either one or two euploid embryos. Twin pregnancies may have resulted. In addition, the hole inserted into the zona pellucida of the blastocyst embryos has had an effect on the timing of the hatching, as stated in the discussion. For that reason, the authors should also include information about the multiple pregnancies (including whether these were monozygotic or dizygotic twin pregnancies).

- We added your point to the result section

Table 2 should also be implemented with an estimate of the miscarriage rate, as PGT-A may lower the incidence of miscarriages. It is of interest, whether the miscarriage rate is indeed different in one of both strategies.

- We changed the term from ‘spontaneous abortion’ to ‘miscarriage’ in table 2

Figure 1 should also have some indication of the statistical analysis (such as p-values). As the reviewers requested: confidence intervals were given.

- We have corrected your point

One statement in the Introduction is misleading to me: why should the timing of cryopreservation and biopsy in the blastocyst stage interfere with genome activation and epigenetic imprinting? I would transfer lines 64 to 67 to line 57, after the first sentence. This sequence of statements is more logical.

- We have corrected your point

Although languagewise the manuscript has been improved, the first few sentences of the discussion must be improved. For example:
Line 29: Abstract: the sentence should be: “Potential use of preimplantation genetic testing for aneuploidy (PGT-A) is increasing.”
Line 126: zona pellucida instead of zona pellucid.
Line 207: lower pregancy rates
Line 239: data not shown instead of data not seen
Line 241: frequently often: remove one of both words

- We have corrected your point.

Reviewer #2: Authors have addressed my comments in the revised version and I do not have any other issues with the manuscript.
